# Free Open Access Medical Education (FOAMed) use in medical students: a literature review

**DOI:** 10.1186/s12909-024-06392-0

**Published:** 2025-01-04

**Authors:** Jacqueline Morgan

**Affiliations:** University of Birmingham, Birmingham, United Kingdom

**Keywords:** FOAMed, Medical, Education, Online, Learning

## Abstract

**Purpose:**

Free Open Access Medical Education (FOAMed) is an emergent phenomenon within medical education. The rise of FOAMed resources has meant that medical education needs no longer be confined to the lecture theatre or the hospital setting, but rather, can be produced and shared amongst any individual or group with access to internet and a suitable device. This study presents a review of the use of FOAMed resources by students as part of their university medical education.

**Method:**

A literature search of terms relevant to the topic of FOAMed use by medical students was completed and reviewed. The included results were subsequently analysed and categorised through qualitative analysis.

**Results:**

The increasingly digital cohort of medical students, fitting into the Gen Z and millennial generations, are generations that have taken strongly to FOAMed resources (Toohey et al*.*, Western J Emerg Med 337–343, 2016, Shorey et al., Nurse Educ Pract 57:103247, 2021), with many of their learning styles being applicable to the methods of study that students were faced with in the online-heavy medical curriculums due to the COVID-19 pandemic (Marshall and Wolanskyj-Spinner, Mayo Clinc Proceedings 95:1135–7, 2020). However, despite the increasing use of FOAMed resources by these students, observed university study recommendations fail to recommend or integrate these resources into the curriculum. This review presents an exploration of the use of FOAMed resources by students as part of their university medical education.

**Conclusion:**

This literature review found that students are increasingly utilising FOAMed as an integral part of their medical education, demonstrating self-determined learning. However, most of the literature on this topic is of the descriptive type, with little literature available on how universities are incorporating this form of student learning into the formal curriculum.

## Introduction

Data suggests that 87% of the population of Great Britain in 2019 was using the internet daily [85], with the use of Social Networking Sites (SNS) being one of the most popular online activities. Amongst those aged 15–25 in the UK, the three most popular SNS sites are YouTube, Facebook, and Instagram respectively [85]. The growth of SNS and Web 2.0, and its design ability of allowing a platform for user generated content has led to the rise of free open access educational resources. This has been a particularly popular avenue for access to medical education resources [18].

Millennials, that is those born between the years 1981–96 [80], have been found to respond to learning that is ‘technology enhanced, convenient, personalised, and linked to relevance and societal meaning’ [47], and medical learning from Free Open Access Medical Education (FOAMed) resources indubitably seems to fit this.

FOAMed is a relatively new term, having its basis in online postgraduate emergency medicine education, with its first usage in 2012 [40]. However, it has been argued that the concept of FOAMed has been present prior to the creation of the term [69, 82]. As such, although the acronym itself gives a self-explanatory understanding of the concept, Nickson and Cadogan [69] offer a broader definition of a ‘dynamic collection of resources and tools for lifelong learning in medicine, as well as a community and an ethos’. From this idea, it follows that any platform that offers this opportunity for dynamic resources and an online community can be counted as one that can offer FOAMed. SNS and Web 2.0 are platforms that can offer this.

FOAMed has utilised most formats available on the internet, including web pages, blogs, SNS posts, and podcasts [21, 46]. Furthermore, it has undergone an evolution, from its earliest pioneers in the early 2000s creating the first free online resources for users, which then began to be shared widely through SNS and the international medical community, to becoming a phenomenon that is increasingly being used by medical organisations, and utilising participation and engagement from the end user [22].

However, there can be an underlying danger to generalising the learning techniques of a whole generation by approaching it purely from this generational theory lens. As Jauregui et al. [51] argue, prior to introducing novel technological resources to a generation grounded on supposed learning stereotypes, particular care should be taken to ensure that the intervention still corresponds to the learning outcomes and considers the specific, unique needs of the individual, which may, in some cases, not align with that of the generation [51, 61]. Jauregui et al. [51], therefore recommends a level of ‘generational humility’ when approaching inter-generational learning needs.

It is unsurprising that medical educationalists have thus taken a keen interest in this growing phenomenon and its impact on medical education, especially for students. Reviews have shown that students tend to respond positively to the emergence of FOAMed resources, however, an objective measure of the effect of the use of these resources on student attainment, and subsequently patient care is often lacking [19, 32, 71].

This review of the literature on FOAMed aims to offer a wide qualitative overview of how FOAMed resources are being used by medical students, with the objective of this to guide further research into the utilisation and incorporation of FOAMed resources into undergraduate medical curricula. For the purpose of this study, FOAMed has been defined as any medical education resource that has been created and/or shared on a SNS or Web 2.0 platform.

### Search strategy

**Fig. 1:**
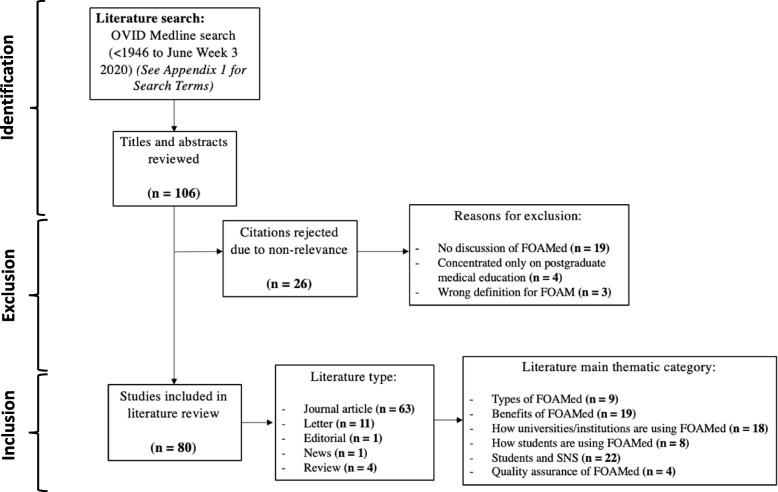
Flow chart demonstrating process for literature review

An English-language literature search was undertaken using search terms relevant to the topic of FOAMed use by medical students (full search strategy can be found in Appendix 1). An initial pilot search was undertaken to gain a scoping overview of commonly used terms and abbreviations that are frequently associated with the topic of FOAMed in the literature, which led to a set of search terms to be used. Search terms included ‘Social media’, ‘Social networking’, ‘web 2’, ‘free open access medical’, ‘FOAM’, ‘FOAMed’, ‘Education, Medical’, ‘Students, Medical’, and ‘Universities’. Titles and abstracts from this search were reviewed for eligibility, and non-relevant results were excluded. Reasons for exclusion included literature that had no discussion of FOAMed, concentrated only on postgraduate medical education, or used a definition of FOAMed that differed from the definition used in this review. This was to ensure that the review maintained its proposed aims and objectives. There were no exclusion criteria for types of literature included, to offer as broad a possible review of FOAMed use in medical students. Literature included journal articles (both qualitative and quantitative), reviews, letters, an editorial, and a news article. Included results were then categorised based on the literature main discussion theme. The process for this literature review, including exclusion criterion, and literature type and thematic categories are demonstrated in Fig. 1.

## Main thematic categories from the literature review

### Benefits of FOAMed

The use of SNS and Web 2.0 as a beneficial addition to medical student education has been widely discussed. From using SNS as a platform for reflective writing [15], to using it to share stigmas within medicine by being able to share personal stories [66], and providing the opportunity to connect with like-minded professionals [8], it is evident that there is no ‘one size fits all’ platform or use of a platform for the purpose of FOAMed. For example, a micro-blogging platform such as X (previously known as Twitter) can offer a platform to directly communicate with and ask questions to others in the field, often giving the opportunity of flattening the medical hierarchy through this accessibility of communication [13], as well as allowing for activities such as ‘virtual journal clubs’ wherein participants can directly communicate with the author,an unheard of concept offline [60, 79]. Whereas, a platform like YouTube, allowing for audio-visual material to be created and disseminated, can allow for the spread of medical education across the globe in a multitude of languages [75]. Or the use of WeChat, a popular social media application in China where most students already have a profile, being used as a way of sharing near-peer communication regarding the hidden curriculum [87, 89].

Nonetheless, regardless of the specificities of the platform or resource utilised, one thing that the advent of FOAMed has managed to unanimously achieve is the globalisation of medical education, where learning is no longer confined to the university or hospital institution [67]. This is especially pertinent for medical education in developing countries [4, 78]. This has allowed for collaborations that have not been possible before, such as a learning intervention between the UK and Somaliland, which utilised a Web 2.0 platform and resulted in two-way learning between the two countries [30]. Further, using an online platform to share educational resources can cut out publication costs, often meaning that these resources can be more accessible [54]. It also has the benefit of ensuring that previously time-limited educational resources are available for a longer period of time. This was evidenced by the intervention of allowing access, via X (previously known as Twitter), of surgical grand rounds, which meant that students could continue to access these learning resources during their revision period [70].

### Types of FOAMed resources

The literature is conflicted regarding the most commonly accessed types of FOAMed platforms and resources by medical students, with some arguing that sites such as Facebook are leading in online medical education [2], whilst others argue that video sharing sites such as YouTube are most popular with medical students [6]. These inconsistencies could be due to the purpose of the resource accessed. For example, students interested in surgery are more likely to access YouTube, as it provides a platform for viewing surgical procedure videos [76], or similarly, the use of YouTube for anatomy education providing a suitable platform for visualisation [49]. Further, platform add-ons such as hashtags (which can be found on sites such as X (previously known as Twitter), Facebook, and Instagram) provide a method for filtering and finding relevant information, thus appealing to certain student online learning requirements. This is particularly useful for students that are interested in a particular specialty, allowing them a way for finding like-minded specialty individuals, who are more likely to post on topics that are of interest to the individual [11]. However, this can be dependent on the particular specialties, as some specialties have been found to have a larger online presence, which may be due to an already established online peer community [83].

A further reason for the lack of consensus with the type of FOAMed platform accessed by medical students is the variance within the researched group of medical students at different stages of their training [41]. For example, pre-clinical medical students may take to platforms that allow for visualisation of concepts, such as video sharing sites, to aid with basic science understanding. Whereas, clinical medical students, who are more involved with hospital based patient education, can be more attracted to platforms that provide the opportunity for collaboration and discussion regarding most up-to-date management. Indeed, as Guraya et al. [41] argue, it is difficult to find a platform that perfectly fits the need for a FOAMed resource due to the heterogeneity of both medical students and medical education. This is further supported by Han et al. [42], where it was argued that the online learning needs of different medical school years differs between pre-clinical and clinical medical students.

### Students and SNS

Again, the literature regarding the incorporation of personal social networking use and medical education is divided. A survey of physician associate (PA) students found that students were, in an informal way, incorporating their social media habits into their education [88]. This is supported by students’ self-reports of using sites such as Facebook to share educational resources between peers [68]. However, a study found that only 25.5% of medical students at a university reported using Facebook for educational purposes [39]. Conversely, other studies have shown higher percentages of student social media incorporation, with values such as 90% [24] and 87.7% [3]. These two latter studies were undertaken across two different countries and cultures – Italy-Romania and Saudi Arabia, suggesting that this is a global phenomenon. On the surface it may appear that there is a stark difference between the quantitative data on student educational incorporation of SNS, however, the difference between the findings in these studies could be due to the definition of SNS. The first study [39] concentrated on Facebook, widely accepted as a SNS, however, the latter two studies did not specify a SNS in their study, with the data including platforms such as Wikipedia and Skype as SNS; platforms that may not be commonly regarded as a SNS [3, 24]. For example, a study of Australian medical students in a rural setting undertaking a specialty rotation, categorised Wikipedia as a Web 2.0 platform rather than an SNS [35]. Therefore, there may be cultural and linguistic differences with certain online definitions, meaning that caution must be taken when extrapolating ideas.

Further studies have found that some students may be less likely to integrate their use of social media into their medical education, despite frequently accessing SNS in their personal time [1, 41, 53]. One of these studies even found that the majority of students find that the use of SNS negatively impacts their academic learning [53]. This is supported by an Irish study, which found that students who self-reported a higher usage of SNS whilst studying, correlated with perceived negative study habits such as ‘cramming’ [10]. However, the difference in these accounts of educational incorporation of social sites, could be due to the institution already incorporating educational resources into SNS, compared to studies where there has been little involvement from the institution of incorporating these sites into the medical curriculum [41]. This difficulty in balancing the positives of utilising personal SNS with its academic potential was identified by Bergl and Muntz [9]. It is suggested that SNS in medical education be utilised to fill in the gaps in the curriculum in a dynamic manner that takes advantage of the platform, rather than presenting already available information on a SNS for the sole purpose of attracting millennial learners [9].

However, Wanner et al. [88] found that the students were willing to engage with medical resources presented on other sites that were not commonly accessed as part of their daily personal usage, such as X (previously known as Twitter). This suggests that students are not necessarily looking for an easy incorporation of resources into their daily habits, but rather, are more interested that the platform for the resource is suitable. Although this study concentrated on PA students, the similarity between the PA and medical course means that these findings may be extrapolated to medical students. Moreover, new additions to a certain platform can introduce a new teaching method, such as the successful incorporation of an X poll (previously known as Twitter) into a geriatrics course [55]. This is despite the study finding that only 33% of medical students used X (previously known as Twitter), compared to 47% of the educators. It also found that none of the students were originally using X (previously known as Twitter) for medical education. This suggests, that again, students are willing to embrace a different social platform from their usual social habits, if the platform and material is fitting to their needs. This is supported by an ethnographic study of medical student X users (previously known as Twitter), which found that the platform was used mainly by these students as a professional tool, with connectivism with the online medical community being a strong factor in their choice of social networking platform [23].

An argument can be made that familiarity on a personal use with a certain site may mean that students are ostensibly more willing to incorporate this site into their medical education [32]. This was argued by Gray, Annabell and Kennedy [40], where it was found that students were more likely to turn to the familiarity of Facebook for their online group studying, despite an online university platform differing very little from the one on Facebook being provided. However, as well as the reasons outlined above regarding platform suitability, other factors may be at play with students’ hesitation in using personal social sites for their medical education. One of the most cited reasons for apprehension for the merging of personal social sites and medical education is the topic of online professional identity. It has been reported that students often receive mixed messages, with some overtly negative faculty attitudes regarding personal SNS use and professional image, leading to confusion and uneasiness with the mixing of these sites and their medical education [44, 56]. Further, students themselves often feel ‘watched’ online, and have been found to find it difficult to completely separate their personal and professional life online [31, 70].

There is also concern that students are not adequately trained regarding formation of their online professional identity [52]. This lack of guidance on their online professional identity may deter students from mixing their personal and medical life online. Indeed, many literature supporting the incorporation of SNS and medical education are aware of this concern [88], with articles arguing for the imperative incorporation of online professional education alongside positive encouragement of using SNS safely [14, 28, 29, 33, 50, 65, 71, 72] Further, Glauser [37] argues that students themselves are mindful of this, with evidence suggesting that students’ are ‘tidying up’ their online presence prior to applying to medical programmes [37]. However, this does not tally with a study that found that 24% of recent medical graduates had completely open settings on their Facebook profile [64]. This is supported by another study which found that 25% of medical student Facebook profiles in one year had some level of open and easily accessible personal information [86]. The difference could, however, be attributed to the year difference, with increasing institutional awareness of online professionalism, and a push from universities to incorporate formal teaching of this in the curriculum [62]. This may suggest that there has been a push regarding online professionalism education in recent years. This is evidenced by a longer study of medical students, starting in 2012 and running for two years assessing the impact of formal teaching of online professionalism, which found a positive change in SNS use after the session, with an increased student awareness of online professionalism [38].

A further consideration for students’ access of FOAMed, apart from the platform itself is the device that it is accessed on. The data has shown that the most popular device to accessing SNS is through a smartphone [24, 58]. However, it has been argued that less than half of students (47.3%) are utilising their smartphone use for educational purposes [63]. This was despite the majority of students form this study (96.8%) admitting to using their smartphone during university-based activities. This raises the concern for distraction whilst using these devices for educational purposes. However, it is to be noted that the data from this study differed between medical students at different levels of their medical education training. For example, it was shown in the data by Loredo e Silva et al. [63] that students in their later years of medical school were more likely to be using their smartphones for accessing educational resources than those in their pre-clinical years. This was especially statistically significant for access to resources that aid in clinical diagnosis and management. This corroborates with the previously discussed study arguing that the type of FOAMed resource access differs between level of study [41]. It may be that smartphones offer a more suitable platform for the FOAMed resources that clinical students are using, for example, wanting quick access whilst on clinical rotations to medical queries. Whereas, pre-clinical students may be more likely to access FOAMed resources for a deeper, or perhaps an initial, understanding of a topic. This is supported in the study by 48.5% of early stage medical students using medical smartphone applications for learning new knowledge compared to 29.9% of late stage medical students. Further, 48.5% of early stage medical students claimed to not have any medical related applications downloaded on their smartphone, compared to 6.6% of late stage medical students [63]. This may mean that as per Guraya et al. [41], the difference is not necessarily due to the different smartphone use habit of students at various stages of their medical education, but rather the types of FOAMed resources that are more easily incorporated into a smartphone.

### How universities/institutions are using FOAMed

However, uncertainties regarding the type of FOAMed platforms and resources that are most beneficial or most used by student have not prevented institutions from incorporating them into the curriculum. Examples of this include specialties utilising social media to recruit medical students [7, 59], using micro-blogging platforms for writing reflections [25], or using Facebook or X (previously known as Twitter) to post regular online questions [84, 87]. However, the majority of institution-based medical student interventions present a form of collaborative discussion platform using popular student SNS [26, 27, 36, 73, 77, 74, 45, 78, 87]. However, there is little robust evidence of the reach and impact of these discussion platforms, bar self-reports and student feedback [27, 43]. Further, with small group discussion sessions moving from a classroom to an online setting, this removes the face-to-face interactions that facilitators have with the students. This raises the concern for online facilitators finding it more difficult to identify struggling students or problems within the group. It may also disadvantage students who are not active users of SNS, or who have reservations of mixing their personal and professional online personas [48, 77].

### How students are using FOAMed

Despite university attempts at integrating SNS and Web 2.0 into the medical school curriculum, there has been an observed trend of dropping student engagement with initiatives that may have been popular in previous years. Hypothesised reasons for this include student frustration with new initiatives on different platforms, unease with sharing thoughts on a topic on such a public platform, and a desire from fee-paying students for more of a personal interaction with their tutors [12]. The latter point is supported by a student letter to the editor advocating for more face-to-face teaching for collaborative subjects, as students are already independently utilising the internet, especially Web 2.0 platforms, for their own understanding of core topics [57]. However, other students have voiced their support for the collaborative benefits of incorporating SNS into their medical education [68]. To narrow the gap between the online education presented by the university and the ones used by students, some institutions have used specially appointed students to guide and share ideas with the university regarding the most suitable and effective method for delivering online education [34]. However, although self-reported support for this is encouraging, there is little empirical evidence of the benefit of these student-faculty relationships.

### Diversity and gender

The literature provides some insight into the usage of FOAMed by different student populations, including gender and country of study. There is discussion regarding the more common types of FOAMed resources used by male or female medical students [1, 3, 6], with some indication that female students preferred platforms that allowed for a more ‘descriptive’ sharing of knowledge [6]. However, when looking at usage of one specific FOAMed resource, the literature suggests very little gender difference with general usage and uptake of the resource [15, 43, 48]. Nonetheless, there was some evidence that there were minor gender differences in how the resource was being used, with some concern that male students were less likely to use resources to ask questions due to a ‘perceived lack of knowledge’ [74]. There was one paper that found that male students benefitted more from a FOAMed initiative on X (formerly known as Twitter), with better test scores [55]. Ramos-Rincón et al. [75] also highlighted a potential risk of female online harassment in these spaces, noting that a student-led video resource had more male viewers, and received many comments of a derogatory nature aimed at the female students in the videos.

It is interesting to note the positive application of incorporating FOAMed resources into formal currucila in developing countries, where technology played a vital role in modern day political revolutions that were largely driven by those from a similar generation [4]. However, Burkholder, Bellows and King [17] found that users accessing FOAMed online resources tended to be from the countries that produced them, with the majority of these being high income countries. They cited reasons for lack of use in lower income countries due to ‘web accessibility and speed, device availability, censorship, and lack of awareness’ [17]. Nevertheless, this has not stopped initiatives for collaborative learning between such countries, with what is called ‘north–south’ and ‘south-north’ teaching [30], as mentioned previously in the section under ‘Benefits of FOAMed’).

It is, however, prudent to note that much of the literature is English language based, and so may not be inclusive of a global overview of FOAMed usage, such as the digital ethnography of X (formerly known as Twitter) medical student users by Chretien et al., [23], which focussed just on English language users. Nonetheless, it is important to note the widespread benefit that platforms with the potential for widespread dissemination can have on increasing the voice of minorities with the medical community, allowing for further diversity and inclusivity [66].

### Quality assurance of FOAMed

A potential reason for the gap between student and university usage of FOAMed resources is the perceived quality assurance of these resources. This is a subject that has been highly debated within the literature, especially within the topic of postgraduate medical education, however, there is little discussion of this in undergraduate medical education. Although there have been applied metrics applied to measure individual resources [83], most individuals use their own critical thinking skills to determine the quality of a resource, which has been argued to be unreliable [81]. This is particularly of concern for medical students, who are using FOAMed resources having not yet completed their medical training. Indeed, early advocates of FOAMed argued that students should cover the bases of a medical education topic by gaining this information from a published textbook, prior to foraying into the online world of FOAMed [69].

For example, a study on a Wikipedia cardiovascular articles found that it was not aimed at a medical audience, and lacked accuracy compared to approved textbooks [5]. Further, there have been concerns regarding sources of FOAMed resources, where the author may have conflicts of interest, such as pharmaceutical companies [16]. Additionally, with an open unregulated platform for the sharing of medical ideas and knowledge, there is the concern of ‘eminence-based medicine’, where those with a stronger online influence may unfairly sway learners, regardless of evidence-base [83].

## Summary

A literature review of the use of SNS and Web 2.0 within medical education argues that the literature presented, regardless of the platform or website used, is a strong demonstration of student self-determined learning [46]. This is a sentiment shared by other studies [20, 78]. As has been demonstrated, the literature regarding the use of FOAMed by medical students is often opposing with regards to which platforms students are utilising, whether they benefit from incorporating their personal SNS use with academic purpose, and how much involvement institutions should have with this type of learning. Part of this could be due to a lack of a concrete definition for FOAMed and what this entails, including what is classed as a SNS and Web 2.0 platform, or it could be due to the heterogeneity of medical students, either due to level of studying or university setting.

However, evidenced by the recent plethora of literature that is now available regarding students and FOAMed, it is clear that FOAMed resources have become a part of modern-day medical student education regardless of usage consensus. This indicates a shift from faculty-led university learning to student self-directed learning. The literature, up to this point, has focused on how universities are incorporating SNS and Web 2.0 into their curriculums, with students’ views on their use of FOAMed resources consisting mainly of quantitative data. Indeed, a systematic review of the use of SNS in medical education found that most literature was still at the ‘descriptive’ phase [19]. Further, there could be more research on how different student populations are utilising FOAMed resources (including whether there are any gender or cultural differences) so as to better tailor these resources more appropriately. Therefore, an exploration of why and how students are using FOAMed resources as part of their medical education, rather than what and when, is needed in order to further understand the lived experience of current medical students and their incorporation of FOAMed resources to their university learning experience.

## Data Availability

No datasets were generated or analysed during the current study.

## Appendix

Database: Ovid MEDLINE(R) <1946 to June Week 3 2020>

Search strategy:

1 exp Social Media/ (7682)

2 exp Social Networking/ (3694)

3 web 2.mp. (27)

4 free open access medical.mp. (22)

5 FOAM.mp. (16514)

6 FOAMed.mp. (266)

7 1 or 2 or 3 or 4 or 5 or 6 (27335)

8 exp Education, Medical/ (164565)

9 7 and 8 (473)

10 exp Students, Medical/ (33736)

11 exp Universities/ (40776)

12 10 or 11 (73712)

13 9 and 12 (106)
